# *C. elegans* colony formation as a condensation phenomenon

**DOI:** 10.1038/s41467-021-25244-9

**Published:** 2021-08-16

**Authors:** Yuping Chen, James E. Ferrell

**Affiliations:** 1 Department of Chemical and Systems Biology, Stanford Medicine, Stanford, CA USA; 2 Department of Biochemistry, Stanford Medicine, Stanford, CA USA

**Keywords:** Emergence, Multicellular systems

## Abstract

Phase separation at the molecular scale affects many biological processes. The theoretical requirements for phase separation are fairly minimal, and there is growing evidence that analogous phenomena occur at other scales in biology. Here we examine colony formation in the nematode *C*. *elegans* as a possible example of phase separation by a population of organisms. The population density of worms determines whether a colony will form in a thresholded fashion, and a simple two-compartment ordinary differential equation model correctly predicts the threshold. Furthermore, small, round colonies sometimes fuse to form larger, round colonies, and a phenomenon akin to Ostwald ripening – a coarsening process seen in many systems that undergo phase separation – also occurs. These findings support the emerging view that the principles of microscopic phase separation can also apply to collective behaviors of living organisms.

## Abstract (web summary)

Phase separation phenomena have emerged as being of critical importance in biology. Here, using colony formation in *C. elegans* as model, the authors demonstrate that the basic concepts that underpin phase separation at a molecular level also apply to collective phenomena at the level of a population of organisms.

## Introduction

Living organisms function both individually and collectively. Examples of organismal collectives range from bacterial biofilms to schools of fish, flocks of birds, and herds of mammals. The likely benefits of such groups include defense from predators and insulation from environmental perturbations. In general, organismal collectives are thought to be able to accomplish things that the individuals cannot^1^.

The formation of a more condensed collective of organisms from a dispersed population breaks the system’s initial spatial symmetry to yield two regions of space—the area containing the collective and the area outside of it—where the organisms have different densities and may have different dynamical properties. In this respect it resembles a phase separation process. Analogous molecular-scale phase separation phenomena include the condensation of water vapor into liquid droplets or the formation of vesicles from dispersed phospholipids; likely examples of biological phase separation include the production of membraneless organelles^2^, such as P-granules^3^, nucleoli^4^, and centrosomes^5,6^ in living cells. Theoretical approaches inspired by the physics of molecular phase separation, including lattice-gas models and reaction-diffusion models, have been successfully applied to a wide variety of collective organismal phenomena, including bacterial flocking and biofilm formation^7^, locust swarming and migration^8^, the formation of spatially intricate colonies by mussels^9^, and the formation of blob-like aggregates or colonies by the annelid *Tubifex tubifex*^10^. The success of these theories underscores the fact there are fundamental similarities between molecular- and organismal-scale phase separation processes, even though the processes take place on very different distance scales, and even though the former involve the passive motions of inanimate molecules whereas the latter involve metabolically-fueled self-propulsion^11–13^.

**Fig. 1:**
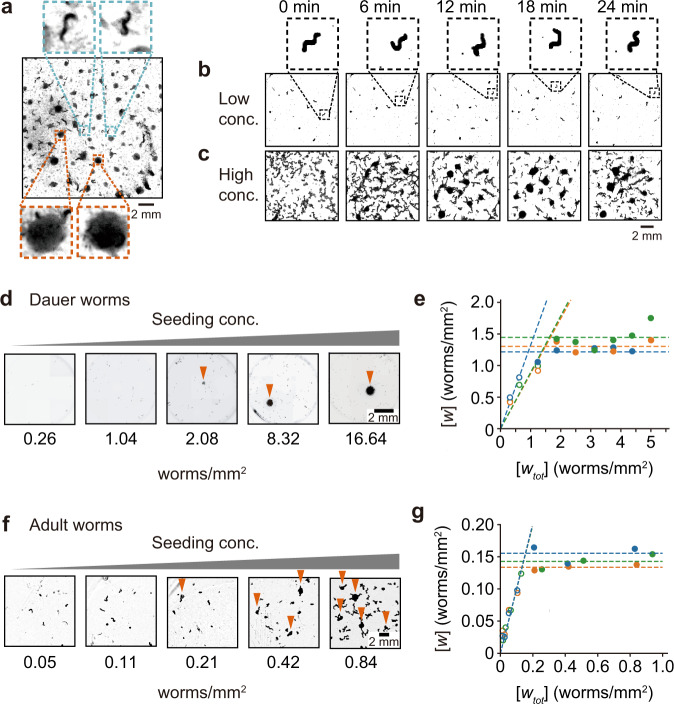
*C. elegans* form colonies at high density in the absence of bacteria. **a** N2 *C. elegans* formed colonies on an NG plate upon consumption of bacteria. Inserts: blue: dispersed worms; orange: colonies. **b**, **c** Washed adult N2 *C. elegans* seeded at a low concentration (0.02 worms/mm^2^) (**b**) did not form stable colonies on an agarose plate (Inserts show individual worms), but those seeded at high density (0.18 worms/mm^2^) (**c**) did. **d**, **f** Dauer-stage worms were seeded on agarose pads (**d**) or adults on agarose plates (**f**) at various densities and the systems were allowed to equilibrate for 30 min. Colonies appeared on pads with higher seeding density (arrows) but not with lower. **e, g** Measured densities of dispersed dauer worms (**e**) or adult worms (**g**) as a function of seeding density. Worms were seeded at various densities on agarose plates and the systems were allowed to equilibrate for 30 min. Plates where colonies formed are indicated with filled circles, and plates with no colonies with open circles. Data are from three independent experiments for either dauer (**e**) or adult worms (**g**).

*C. elegans* is a macroscopic nematode that exhibits chemotaxis^14^, learning^15^, and complex social behaviors^16,17^. In the course of other studies, we observed what appears to be a simple example of colony formation in *C. elegans*: we found that when N2 Bristol worms grow on plates to high density (a high number of worms per unit area), so that the bacterial food source was exhausted, they often formed colonies (Fig. 1a). (Here we will use the term “colony” even though these colonies are not the descendants of a single founding organism the way a bacterial colony or a yeast colony is.).

Here we have examined *C. elegans* colony formation through quantitative experiments and condensation theory. Experimentally we found that colony formation occurs only when worms are plated at a density above a critical value. A compartmentalized ordinary differential equation (ODE) model of the process accounts for this and predicts that the ratio of the rates of colony exit to colony entry determines the critical density. We found this prediction to be true, within experimental error, for both small dauer worms and large adult worms, even though the critical concentrations for the two types of worms differed by an order of magnitude. We also found that when multiple colonies were present, they sometimes fused to form larger colonies, and they also underwent Ostwald ripening, where large colonies grew and small colonies shrank, two coarsening mechanisms that are also seen in molecular phase separation processes. These findings indicate that *C. elegans* colony formation can be regarded as a phase separation phenomenon, and provide support for the idea that the basic principles of condensation and phase separation apply across a wide range of distance scales.

## Results

### *C. elegans* colony formation

Previously it was shown that some strains of *C. elegans* feed in clumps at the edges of existing bacterial lawns^18^. This social feeding behavior was attributed to the sensing of local oxygen levels^19,20^. However, some strains, including the classic lab strain N2 Bristol, lack the ability to clump in this fashion^18^, but still do form colonies under some conditions (Fig. 1a). Recently, Demir et al. reported what appeared to be a related patterning behavior, and showed that it depended on bacteria^21^.

To test if the formation of *C. elegans* colonies shown in Fig. 1a requires bacteria, we grew N2 worms to adulthood and then washed them and transferred them to a fresh agarose plate in the absence of added bacteria. A high density (0.18 worms/mm^2^) of adult worms formed colonies within minutes (Fig. 1c, Supplementary Movie 1), whereas a low density (0.02 worms/mm^2^) did not (Fig. 1b, Supplementary Movie 2). Even after 12 h of incubation, low densities of worms did not form colonies (Supplementary Fig. 1). These findings suggest that colony formation depends upon having a sufficient density of worms, but does not require the presence of bacteria or of a pre-existing pheromone pattern.

We also tested *C. elegans* at other developmental stages, including dauer and asynchronous stages. Notably, to obtain dauer worms (small larvae that have been induced to enter an alternative developmental stage in which they are resistant to harsh conditions), we washed and treated with a low dose of detergent before replating, which should lyse and eliminate any trace amounts of bacteria. As was the case with adults, worms in these developmental stages formed colonies when plated at high density, but not at low density (Supplementary Fig. 2). Once again, a system consisting of worms alone, with no bacteria, was able to undergo colony formation, and this colony formation depended upon the density of the worms.

### Critical densities in dauer and adult worms

We then looked in more detail at the density dependence of colony formation. We placed different densities of dauer-stage N2 worms on an agarose plate, gently spread the worms, and took pictures of the plates after 30 min, a time when pilot experiments showed that the colonies were stable and no further colonies were forming. Once again there was a threshold for colony formation (Fig. 1d, e): a stable colony only appeared when the seeding density was above the critical density, and all conditions above the critical seeding density resulted in stable colonies. Moreover, the density of the out-of-colony worms increased linearly with the seeding density when the seeding density was below the threshold, and attained a constant maximal value when the seeding density exceeded the threshold (Fig. 1e). The critical density was 1.33 ± 0.25 worms/mm^2^, estimated by fitting a straight line to the data points where there were no colonies (open circles, Fig. 1e), a flat line to those data points where there were colonies (filled circles, Fig. 1e), calculating the intersection between the two lines, and then averaging over three independent experiments. Thus, there is a critical density requirement for the formation of dauer-stage *C. elegans* colonies, and above this density, colonies and dispersed worms coexisted.

To test if the thresholded response of colony formation is stage specific, we performed the same experiment with adult worms (Fig. 1f, g). Again the density of out-of-colony worms rose approximately linearly until the critical density was reached, and then remained constant. However, the critical density for adult worms (0.148 ± 0.016 worms/mm^2^) was almost an order of magnitude smaller than that of dauer larvae.

### *C. elegans* dynamics on second-to-minute time scales

**Fig. 2:**
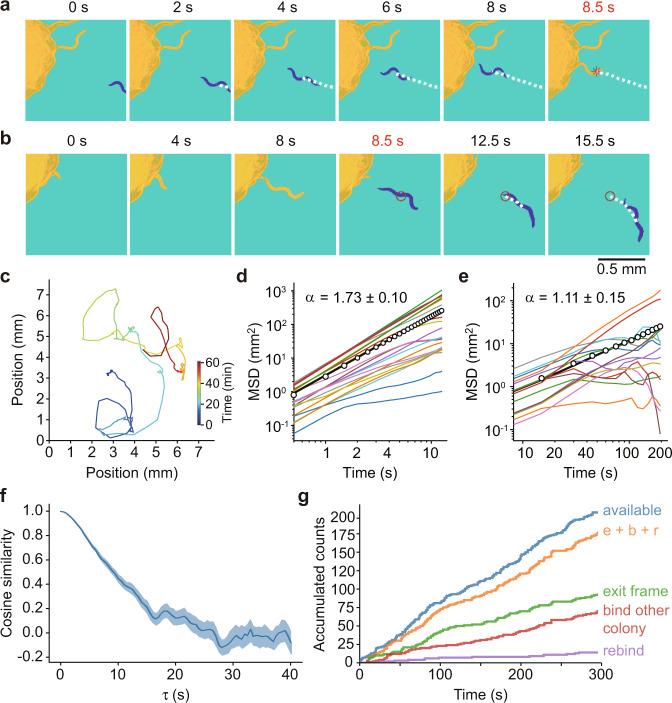
*C*. *elegans* moves ballistically on second time scales and in a random walk fashion on longer time scales, and rarely returns to previous colonies. **a** A montage of a worm entering a colony. False coloring and marks were generated by an automatic tracking algorithm. Yellow: a portion of colony; blue: an out-of-colony worm; white dashed line: trajectory; red asterisk: algorithm-called entering event. **b** A montage of a worm exiting a colony. Coloring and labels are the same as in **a**. Red open circle: algorithm-called exiting event. **c** Sample trajectory of a single worm. **d**, **e** Mean squared displacement (MSD) vs. time interval for 15 individual worm trajectories, plotted on log–log plots. The data in the two panels are from separate independent experiments. The open circles represent ensemble MSDs for the binned data and the thick black lines are straight-line fits, which yielded values for α, the log–log slope, of 1.73 ± 0.10 (*r*^2^ > 0.9999), and 1.11 ± 0.15 (*r*^2^ = 0.9990), respectively. **f** Cosine similarity between the direction of *C. elegans* movement at times *t* ant *t* + τ, as a function of the value of τ. The blue center line indicates the median value across 432 traces from 4 independent experiments. The blue shaded area indicates the 95% confidence interval for the median value. **g** Ultimate fates of the worms that left colonies from eight imaging experiments. The cumulative total number of worms to have left a colony is designated “available” (blue). The other four colors represent the number of worms that rebound the same colony (purple), bound a different colony (red), exited the frame (green), or did any of these three things (e + b + r, orange). The number of worms that remained dispersed and still in frame is represented by the difference between the blue and orange curves.

To begin to understand why a critical density of worms was required for colony formation, and what determined the value of the critical density, we set out to quantitatively characterize the worms’ dynamics. In particular, we addressed three questions: (1) Were the worms’ movements ballistic, with their displacement from a starting point being directly proportional to time, or were they random walks, with the square of the displacement proportional to time? (2) Did the worms move differently depending upon how close they were to a colony, as is the case with chemotaxis? And (3) did the worms slow down when they joined a colony? To this end, we developed a tracking algorithm to automatically record the movement of individual dauer worms as well as their interactions with colonies. The algorithm allowed tracking of the trajectories of multiple worms (Fig. 2a, b; Supplementary Movie 3), and identified when a worm entered (Fig. 2a) or exited (Fig. 2b) a colony.

On a time scale of minutes, the trajectories of the out-of-colony worms resembled a random walk^22^ (Fig. 2c), with the worms alternating between time periods when they crawled along fairly straight trajectories and periods when they stopped and turned. A log–log plot of mean squared displacement vs. time from 15 to 195 s yielded a slope of α = 1.11 ± 0.15 (Fig. 2e), close to the expected value for a random walk (α = 1). In contrast, over the first few seconds the trajectories were more ballistic. A log–log plot of mean squared displacement vs. time from 0.5 to 13 s yielded a slope of α = 1.73 ± 0.10 (Fig. 2d), closer to that expected for a ballistic trajectory (α = 2). Thus, on a time scale of a few seconds the worms’ trajectories were approximately ballistic, with mean displacement being directly proportional to time, whereas on a time scale of tens of seconds, the trajectories were close to random walks, with mean squared displacement being proportional to time.

To further characterize the motion, we examined how quickly the direction of a worm’s trajectory became uncorrelated, by calculating the cosine-similarity angular autocorrelation function (Fig. 2f). Trajectories lost their directional autocorrelation by 30 s, and the half-time for the loss was 9.3 s (Fig. 2f). The distance a worm traveled during this time interval is on the same order as the size of a colony (0.1–1 mm). This shows again that on a time scale of ~10 s or longer we can regard the worms’ trajectories as being random walks, and on shorter time scales the trajectories are more ballistic.

Ballistic departure of a worm from a disk-shaped colony, with little rebinding (re-entering the colony it originated from), would make it possible to formulate a relatively simple model for the dynamics of the worms. We therefore directly addressed whether rebinding contributes substantially to worm dynamics, by measuring the frequency at which worms returned to their original colony after leaving (Fig. 2g). In this analysis, a departing worm becomes available for four classes of action: (i) rebinding (Fig. 2g, purple); (ii) binding another colony (Fig. 2g, red); (iii) exiting the frame (Fig. 2g, green); or (iv) remaining out-of-colony. We found that only a small fraction of worms returned to the original colony (7%) compared to worms joining a different colony (31%), exiting the frame (42%), or remaining out-of-colony (20%). This indicates that the worms almost always made it into the bulk medium once they left a colony. Note that in theory this may not be true if the colonies grow to too large a size, but for the experiments shown here rebinding was infrequent. The assumption of negligible rebinding will help simplify the modeling described below.

### The *C. elegans* trajectories are not biased toward colonies

At least two classes of mechanism might explain the formation of colonies at high worm densities. First, colony formation could be due to chemotaxis (or some other form of taxis), with the worms moving preferentially toward a nascent colony in response to a gradient of an attractant. In this taxis model, worms would move at different speeds, or with different directional persistence, depending upon whether they were moving toward the colony or not. Bacterial chemotaxis, where bacteria sense and move up a concentration gradient of a chemo-attractant like serine or aspartate, is an example of this type of mechanism^23–25^. Alternatively, the worms could be moving randomly when in the dispersed phase, but then become captured by a colony if they accidentally collide with it because the colony slows them down. This random capture model would be more like what happens in a molecular condensation process like the condensation of water vapor or the formation of micelles from dispersed detergent molecules.

**Fig. 3:**
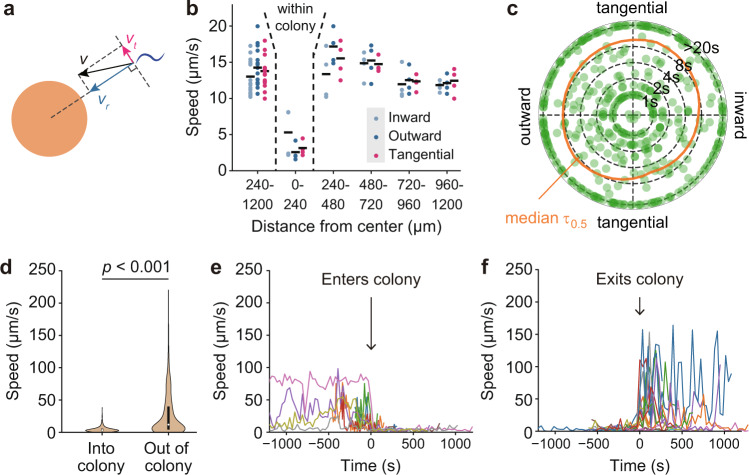
Chemotaxis vs. random capture. **a** Schematic of breaking down a worm’s velocity into radial and tangential components. A colony is shown by the orange disc, and a worm by the blue curl. The radial component of the velocity is shown as a blue arrow, and the tangential component as magenta arrow. **b** Measured radial and tangential velocities for worms overall and at various distances from a colony. Most of the worms in the 0–240 µm bin were in colonies. The data for worms at distances outside of this bin are shown both separated by bin (240–480, 480–720, 720–960, and 960–1200 µm) and in aggregate (240–1200 µm). Data points are average velocities for worms in each bin, for each of 4 independent experiments done on 2 days. The total number of velocities averaged together for each data point ranged from 10,604 to 45,530. Velocity components were averaged for each colony at each distance before statistical analysis. A two-sided, Shapiro test failed to reject normality in all data bins; *p*-values were 0.74–0.99. *p* values for comparison of the inward, outward, and tangential components of the velocities were calculated by two-sided Student’s *t*-test and ranged from 0.23 to 0.91 for the four bins between 240 and 1200 μm. **c** The time for the cosine similarity to drop to 0.5 (τ_1/2_) for individual worms with different initial orientations. On this polar coordinate representation, the radius denotes the τ_1/2_ and the angle denotes the angle between the worm’s initial trajectory and the center of the colony, with due east being 0°. Median τ_1/2_ values were calculated for worms binned into six wedge-shaped angular regions, and a smooth spline curve was fitted to the median values (orange). **d** Violin plots of the speeds of worms in colonies and outside of colonies. The black bars span the 25–75th percentiles. Open circles indicate median values. The Wilcoxon rank-sum test *p*-value is shown (two-sided; *n* = 769 in-colony; 1378 out-of-colony, *p* = 5 × 10^−91^). **e**, **f** Examples of worms slowing down upon entering a colony (**e**) and speeding up upon exiting a colony (**f**).

Thus, we examined whether the velocities of the worms were different in different directions, and if they were different close to a colony vs. far from a colony. We measured the radial and tangential components of velocities of worms at different distances from the center of a colony (Fig. 3a). If a worm were attracted to a colony, one would expect the radial component to be greater than the tangential component. However, we did not see such a trend (Fig. 3b; Wilcoxon *p*-value = 0.73 for the overall radial vs. tangential comparison). We also dissected the radial and the tangential components of the velocity after binning the worms according to their distance to a colony (Fig. 3b), in case some difference might be more apparent close to a colony. At all distances from the colony, the radial velocity was similar to the tangential velocity, and no significant difference between the speeds of worms moving toward vs. away from the colony was found (Fig. 3b). Finally, we determined whether the directional persistence of the worms’ trajectories differed depending upon whether they were moving toward or away from a colony. We plotted the half-times for losing directional autocorrelation as the *r*-coordinate on a polar coordinate plot, and plotted the worms’ directions as θ (Fig. 3c). The half-times were unchanged regardless of the worms’ directions. These findings argue against the taxis model.

Alternatively, we measured the speed of worms in colonies vs. outside of colonies to see whether in-colony-slowing, due to their interactions with other worms, might account for colony formation. The second bin in Fig. 3b (0–240 µm from the colony center) consists largely of in-colony worms, and their average velocities were substantially lower than those of worms in the more distant bins, suggesting that worms in the colonies do move more slowly than dispersed worms. To further quantify the change in velocity, we tracked the movements of fluorescently labeled worms sparsely mixed with label-free worms (Supplementary Movie 4). On average, worms outside of the colony moved six times faster than worms in a colony (Fig. 3d) (Wilcoxon *p*-value < 0.001). This spatially distinct behavioral difference could be the result of (i) two behaviorally differentiated populations of worms, or (ii) a single population of worms that switched rapidly between two behavioral states. To distinguish between these possibilities, we tracked individual worms before and after they transited into or out of a colony. We found that individual worms promptly accelerated upon leaving a colony and decelerated upon joining a colony, supporting the hypothesis that the worms in and out of the colonies were a single population with two behavioral states (Fig. 3e, f).

These findings support the hypothesis that a distribution of essentially identical individual worms forms and maintains colonies through a mechanism where the worms randomly collide with and join colonies, and then slow their movement.

### A two-compartment ODE model of worm dynamics

**Fig. 4:**
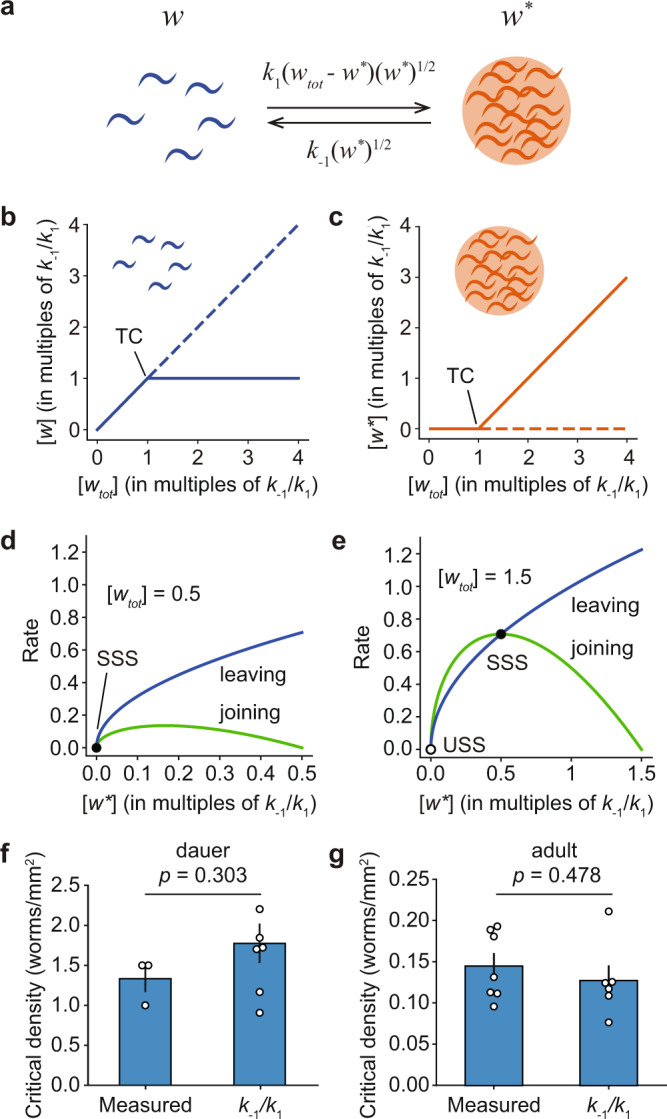
A simple two-compartment model accounts for thresholded *C. elegans* colony formation. **a** Schematic of the rate equation model for colony formation. **b**, **c** The modeled steady-state densities of worms out of colonies (*w*), and in one or more colonies (w*), as a function of the total density of worms *w*_*tot*_, based on Eqs. 7 and 8. The system has a single stable steady state until the concentration of worms reaches a critical value of *w*_*tot*_ = *k*_−1_/*k*_1_ (TC, transcritical bifurcation point). Beyond the critical density, the system bifurcates, and has an unstable steady state (dashed) and a stable steady state (solid) with a constant density of the dispersed worms *w*. **d**, **e** Rate-balance analysis of a case with *w*_*tot*_ smaller than the critical density (*w*_*tot*_ = 0.5*k*_1_/*k*_−1_), and one with *w*_*tot*_ greater than the critical density (*w*_*tot*_ = 1.5*k*_1_/*k*_−1_). The joining (green) and leaving (blue) rates intersect at the steady states. In **d**, the single steady state with w* = 0 is stable (SSS), as small perturbations make leaving occur faster than joining, making the system return toward the steady state. In **e**, the steady state at w* = 0 is unstable (USS), as small perturbations would make joining occur faster than leaving, and drive the system away from that steady state. **f**, **g** Critical density of dauer worms (**f**) and adult worms (**g**) measured directly as in (Fig. 1e, g) (mean ± S.E.M.; *n* = 3 for dauer and *n* = 7 for adult) and compared to predicted critical density values obtained by measuring *k*_*−1*_ and *k*_*1*_ from time-lapse movies of dauer-stage worms (*n* = 7) or adult worms (*n* = 6) (not necessarily at steady state). Measurements were taken from distinct samples. The *p* values were calculated using student’s *t*-test (two-sided, Shapiro normality test failed to reject normality).

Since the worms’ velocities depended strongly upon whether they were in or out of a colony, but not on their specific positions, we chose to model the dynamics of the worms with compartmentalized ODEs. We considered all of the out-of-colony worms (designated *w*) to be in one well-mixed compartment, with a uniform density of *ρ* = *w*/*A*, where *A* is the area of the agarose plate or pad, and all of the in-colony worms (designated w*) to be in another compartment, with a higher density of ρ* = w*/A*, where A* is the area of the colony (Fig. 4a). Initially we will assume that there is a single colony, as was the case in some of the experiments we carried out (e.g., Fig. 1d). Later we will consider multiple colonies.

The rate at which the out-of-colony worms (*w*) collide with and join a circular colony should be proportional to the number of out-of-colony worms that happen to be at the boundary of the colony. If the length of the boundary is 2*πr*, where *r* is the radius of the colony, then:

1 $${{{{{\mathrm{Joining}}}}}}\,{{{{{\mathrm{rate}}}}}}\propto r\cdot w.$$

The radius of the colony is assumed to be proportional to the square root of its area and the square root of the number of worms in the colony, w*. Thus:

2 $${{{{{\mathrm{Joining}}}}}}\,{{{{{\mathrm{rate}}}}}}={k}_{1}w{(w{\ast} )}^{\frac{1}{2}},$$

where the rate constant *k*_1_ is determined by the speed of the out-of-colony worms and the fraction of the trajectories that aim into the colony (ideally 0.5). Note that this assumes that the persistence length of the motion is long compared to the diameter of the colonies. In actuality, the two length scales are similar (Figs. 2, 3); nevertheless, we start with this assumption because it simplifies the analysis.

Similarly, we assume that only the in-colony worms that are on the circumference of a colony can exit it. Thus, the rate at which worms leave a colony is again proportional to the square root of the area of the colony ${(w{\ast} )}^{\frac{1}{2}}$:

3 $${{{{{\mathrm{Leaving}}}}}}\,{{{{{\mathrm{rate}}}}}}={k}_{-1}{(w{\ast} )}^{\frac{1}{2}}.$$

The rate constant *k*_−1_ is determined by the speed of the in-colony worms and the fraction of the trajectories that aim out of the colony (again, ideally 0.5).

The net rate of colony formation can therefore be written as:

4 $$\frac{dw\ast }{dt}={k}_{1}w{(w{\ast} )}^{\frac{1}{2}}-{k}_{-1}{(w{\ast} )}^{\frac{1}{2}}.$$

Experimentally we found that less than 1–3% of the worms crawled off the agarose pads in the typical 30–45 min experiment. Thus, we assume that the total number of worms in the system is a constant *w*_*tot*_, which means that:

5 $${w}_{tot}=w+w{\ast} .$$

Therefore, Eq. 4 can be written as:

6 $$\frac{dw{\ast} }{dt}={k}_{1}({w}_{tot}-w{\ast} ){(w{\ast} )}^{\frac{1}{2}}-{k}_{-1}{(w{\ast} )}^{\frac{1}{2}},$$

with a single time-dependent variable (w*). This is the compartmentalized one-ODE model for the equilibration of worms between a dispersed phase and a colony. Note that there is positive feedback in this model, since the larger w* is, the faster the joining rate will be, at least until w* becomes a substantial fraction of *w*_*tot*_.

For the system to be in steady state, the time derivative must equal zero:

7 $$0={k}_{1}({w}_{{{{{{\mathrm{tot}}}}}}}-w_{ss}{\ast}){(w_{ss}{\ast})}^{\frac{1}{2}}-{k}_{-1}{(w_{ss}{\ast})}^{\frac{1}{2}}$$

where ${w}_{ss}{\ast }$ is the steady-state number of worms in the colony. There are two solutions for ${w}_{ss}{\ast }$:

8 $${w}_{ss}{\ast }=0$$

which means that there is no colony and all of the worms are dispersed and solitary, and:

9 $${w}_{ss}{\ast }={w}_{{{{{{\mathrm{tot}}}}}}}-\frac{{k}_{-1}}{{k}_{1}}.$$

Note that neither ${w}_{ss}{\ast }$ nor *w*_*tot*_ can be smaller than zero. This means that Eq. 9 provides a physically meaningful solution only if *w*_*tot*_ > *k*_−1_/*k*_1_. There is a single steady state when *w*_*tot*_ ≤ *k*_−1_/*k*_1_, given by Eq. 8, and two steady states when *w*_*tot*_ > *k*_−1_/*k*_1_ (Fig. 4b, c), given by Eqs. 8 and 9.

To determine which of the steady states is stable, we performed rate-balance analysis (Fig. 4d, e). When *w*_*tot*_ ≤ *k*_−1_/*k*_1_, the single steady state, with ${w}_{ss}{\ast }=0$, is stable, since perturbing the system from this steady state makes the leaving rate from the colony become faster than the joining rate, pushing the system back to the ${w}_{ss}{\ast }=0$ steady state (Fig. 4d). On the other hand, when *w*_*tot*_ > *k*_−1_/*k*_1_, and there are two steady states, the one with ${w}_{ss}{\ast }={w}_{{{{{{\mathrm{tot}}}}}}}-\frac{{k}_{-1}}{{k}_{1}}$ is stable, and the one with ${w}_{ss}{\ast }=0$ is unstable (Fig. 3e). Thus, when *w*_*tot*_ is below a critical value of *w*_*tot*_ = *k*_−1_/*k*_1_, no colony will form, and any pre-existing colony will disperse. And when *w*_*tot*_ is above this critical value, a colony will form, with the size of the colony depending upon how far above the critical value *w*_*tot*_ is and the density of the out-of-colony worms remaining at its maximal possible value of *k*_−1_/*k*_1_ (Fig. 4b–e). The transition from one to two steady states that occurs at *w*_*tot*_ = *k*_−1_/*k*_1_ is termed a transcritical bifurcation^26^. Transcritical bifurcations are seen in simple models of micelle formation, liquid–liquid phase separation, and precipitation, various condensation processes that occur on a molecular level^27–29^.

Note that in this model, the velocities of the worms in the colony must be slower than the velocities of the out-of-colony worms, as was found experimentally (Fig. 3), if the colony is to be stable. The maximum inward flux across the boundary (the joining rate) depends upon the maximum overall density of the dispersed worms (which is approached when the colony is infinitesimal) times their speed; the outward flux depends on the density of the condensed worms times their speed. Thus, if the density of the worms is higher in the condensed phase than it is in the dispersed phase, then the velocities in the condensed phase must be slower (and by a factor at least as big as the ratio of the densities) than they are in the dispersed phase, or there will be a net outward flux and the condensed phase will not be stable. In this respect—the requirement that the worms slow down in the condensed phase—the model is similar to mobility-induced phase separation models^11–13^, although that class of model obtains its positive feedback from a relationship between density and velocity, rather than between the size of the condensate and the joining rate.

Note also that so far we have considered the interplay between dispersed worms and a single colony. However, the analysis can easily be extended to multiple colonies, provided that the values of *k*_−1_ and *k*_1_ do not vary with colony size (Supplemental Text), and the analysis yields the same prediction of a critical worm concentration *w*_*tot*_ = *k*_−1_/*k*_1_ below which colonies will not form.

Thus the compartmentalized ODE model of condensation by random capture predicts that (i) at steady state, there will be a density threshold, above which one or more colonies form, and below which no colony forms; (ii) when the seeding density is above the colony formation threshold, the steady-state density of out-of-colony worms should be constant; and (iii) the critical colony concentration is equal to the ratio of the association and dissociation rate constants, *k*_−1_/*k*_1_.

### Testing the model’s predictions

According to the model, the colony-forming threshold should equal *k*_−1_/*k*_1_. This ratio can be determined experimentally from three measurable quantities: the rate at which worms leave the colony, the rate at which worms enter the colony, and the concentration of out-of-colony worms:

10 $$\frac{{{{{{\mathrm{Leaving}}}}}}\,{{{{{\mathrm{rate}}}}}}}{{{{{{\mathrm{Joining}}}}}}\,{{{{{\mathrm{rate}}}}}}}=\frac{{k}_{-1}{(w{\ast} )}^{\frac{1}{2}}}{{k}_{1}w{(w{\ast} )}^{\frac{1}{2}}}=\frac{{k}_{-1}}{{k}_{1}}\frac{1}{w},$$

11 $$\frac{{k}_{-1}}{{k}_{1}}=w\cdot \frac{{{{{{\mathrm{Leaving}}}}}}\,{{{{{\mathrm{rate}}}}}}}{{{{{{\mathrm{Joining}}}}}}\,{{{{{\mathrm{rate}}}}}}}.$$

We took time-lapse videos of worms near existing colonies and measured the association and dissociation rates and the out-of-colony worm density (Supplementary Movie 3). For dauer worms, the estimated value of *k*_−1_/*k*_1_ from these measurements was 1.78 ± 0.25 worms/mm^2^ (mean ± S.E., *n* = 7), somewhat higher than the directly measured threshold of 1.33 ± 0.17 worms/mm^2^ (mean ± S.E., *n* = 7). Given the experiment-to-experiment variation, this difference is not statistically significant (Student’s *t*-test *p*-value = 0.303) (Fig. 4f). For adult worms, the predicted threshold was an order of magnitude lower (0.130 ± 0.019 worms/mm^2^, mean ± S.E., *n* = 6), again in reasonable agreement with the directly measured threshold (0.148 ± 0.016 worms/mm^2^, mean ± S.E., *n* = 7) (Student’s *t*-test *p*-value = 0.478) (Fig. 4g). If we take Joining rate*/w* as a gauge of *k*_1_, and Leaving rate as a gauge of *k*_−1_, the adult worms join colonies ~2× faster and leave colonies ~7× slower than dauer. Adult worms appear to be held more tightly by a colony than dauers are, perhaps because their larger size affords them more physical interactions with other worms. As predicted by the ODE model, changes in the dynamics of joining and leaving results in a change in the critical density.

Thus, the compartmentalized, random capture ODE model both qualitatively and, within error, quantitatively accounts for the thresholds in *C. elegans* colony formation for dauer and adult worms.

### Spatial coarsening

**Fig. 5:**
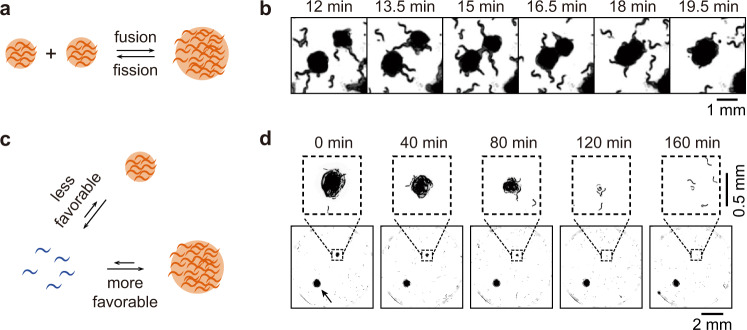
Spatial coarsening during colony formation. **a** Fusion of two small colonies to form a larger one. In principle colony fission might occur as well. **b** An example of colony fusion, from Supplementary Movie 1. **c** A simple model for Ostwald ripening, where the *k*_−1_/*k*_1_ is assumed to decrease with colony size. **d** An example of a small colony that progressively decreases in size and then disappears.

In many inhomogeneous physical–chemical systems, the small structures shrink over time and eventually disappear, while the large structures grow. Several mechanisms can contribute to this coarsening. One is the fusion of less-stable small colonies to form more-stable larger colonies (Fig. 5a), which has been found to occur in *Tubifex tubifex* blob formation^10^. Under conditions where multiple *C. elegans* colonies form, this type of coarsening occurred. One such example is shown in Fig. 5b: a single worm makes simultaneous contact with two approximately circular colonies, which then coalesce into a larger, single, circular colony.

When colonies were too far apart to make physical contact with each other, we found another type of spatial coarsening—Ostwald ripening, where large colonies grow at the expense of smaller ones through competition for worms exchanged into the bulk, dispersed phase^30,31^. Like fusion, Ostwald ripening is driven by greater stability of the larger structures. An example of Ostwald ripening is shown in Fig. 5d. A small modification to the rate equation model (Eq. 5)—adding size dependence to the colonies’ stability—is sufficient to generate Ostwald ripening in the worm model (Fig. 5c; Supplementary Text and Supplementary Fig. 3).

## Discussion

We have demonstrated that at high density *C. elegans* can self-organize and form colonies, even in the absence of bacteria. The worms move essentially randomly on agarose, and if they encounter a small colony they can join it. As the colony grows, it becomes a bigger target for the addition of more worms, which provides the process with positive feedback—the rate of colony joining increases as the number of worms that have already joined increases. Worms in a colony move more slowly than dispersed worms do, which allows the colonies to be stable structures.

Even though this is a complex behavior exhibited at the level of a group of living, self-propelled organisms, *C. elegans* colony formation can be explained by a simple model that could also be applied to passive processes like precipitation or micelle formation. The model predicts a density threshold for colony formation and a constant, maximal density of out-of-colony worms when the threshold is reached. We found these predictions to be correct through direct experimental observation. With small modifications, the model can account for the phenomenon of Ostwald ripening as well.

Since the dispersed worm phase conforms to the size and the shape of the dish or pad the worms are plated on, it behaves like a vapor phase. The colonies can be viewed as a liquid phase: their size is determined by the number of worms in the colony, they are usually roughly circular, suggestive of surface tension, they can fuse, and the worms within the colony are moving rather than frozen in place. Thus, *C. elegans* colony formation may be viewed as analogous to the condensation of a vapor into a liquid, even though it is not being driven by thermal motions and free energies.

The physics of molecular phase separation is a well-developed field, and there are theories to account for aspects of the process that are not considered in the compartmentalized ODE model presented here. For example, the Smoluchowski coagulation model^32^, which is a rate equation approach like the ODE model used here, explicitly allows for the possibility that the dynamics of the addition of a molecule to an *n*-molecule condensed phase differs depending upon the value of *n*. Many modern treatments of phase separation at both the molecular level and the organismal level are based on partial differential equations, like the Cahn-Hilliard equation, that include spatial terms to describe mass transfer as well as reaction terms based on an assumed chemical potential function for the condensation process. Particularly notable are the motility-induced phase separation models, theories that have emerged from active matter physics and which have been successfully applied to biological condensation processes as well^11,13^. Such treatments predict that over some time range, the growth of the condensed phase will obey a power law relationship. We have not yet been able to conclusively rule in or out the expected power law scaling. However, Deblais and co-workers^10^ were able to show that in what they termed “blob” formation by *Tubifex tubifex* annelids placed in water, a power law relationship between colony size and time was indeed observed.

That said, it is perhaps remarkable how well the simple compartmentalized ODE approach works. Moreover, the ODE approach makes it easy to appreciate the basic concepts that underlie phase separation—the positive feedback that arises because the colony’s joining rate depends upon how many worms have already joined, and the slowing of the worms once they have joined. These concepts are shared by more sophisticated theoretical approaches^11–13^.

Together with other recent work^7–10,21^, these observations indicate that biological self-organization and pattern formation, through phase separation, occurs across many scales, from molecules^33–36^, organelles^3,37^, and possibly subcellular compartments^38^, all the way to a population of organisms.

## Methods

### Strains and maintenance

*C. elegans* strains, N2 Bristol and isogenic GFP-labeled GA631, and *E. coli* OP50 were obtained from the Caenorhabditis Genetics Center, University of Minnesota. *C. elegans* were maintained and cultured routinely on nematode growth (NG) plates according to standard procedures^39^.

### Liquid culture

To obtain sufficient numbers of *C. elegans*, we cultured *C*. *elegans* in liquid before experiments. *C. elegans* from a 100 mm plate that had been just depleted of food were washed and transferred to 250 mL S Medium^40^ (1 L autoclaved S Basal plus 10 mL 1 M potassium citrate pH 6, 10 mL trace metals solution, 3 mL 1 M CaCl_2_, 3 mL 1 M MgSO_4_. S Basal: 5.85 g NaCl, 1 g K_2_HPO_4_, 6 g KH_2_PO_4_, 1 mL cholesterol (5 mg/mL in ethanol), H_2_O to 1 L; trace metals solution: 1.86 g disodium EDTA, 0.69 g FeSO_4_•7H_2_O, 0.2 g MnCl_2_•4H_2_O, 0.29 g ZnSO_4_•7H_2_O, 0.025 g CuSO_4_•5H_2_O, H_2_O to 1 L) with 0.5 mL *E. coli* OP50 pellet. To obtain adult worms, cultures were shaken in flasks and incubated at 23 °C for 3 days with monitoring of bacterial density to ensure no starvation of worms. Adults were enriched by transferring cultures to 50 mL Falcon tubes, settled for 5 min, and collected from the bottom. The adults were then washed and settled twice in fresh M9 buffer^40^ (3 g KH_2_PO_4_, 6 g Na_2_HPO_4_, 5 g NaCl, 1 mL 1 M MgSO_4_, H_2_O to 1 L). To obtain dauer-stage worms, the liquid culture was maintained similarly except cultured for 10 days. Dauer stage was induced by the high population density in the culture. Worms were transferred to conical 50 mL tubes, and collected from the bottom of tubes after centrifugation at 300 × *g* for 3 min. To remove worms in other developmental stages, worms were then resuspended and incubated in 1% SDS for 40–60 min at room temperature. To separate dauer worms from debris, material was then collected by centrifugation at 300 × *g* for 3 min and followed by centrifugation at 16,000 × *g* for 1 min at 4 °C in a tube containing cooled M9 buffer on top and 30% sucrose in M9 buffer at the bottom (sucrose cushion floatation). Dauers were enriched at the interface between the two density layers after centrifugation. These worms were quickly collected with a wide-bore Pasteur pipette and washed twice with M9 buffer at room temperature. Culture density was estimated by counting 20 μL droplets of the purified cultures (or a larger volume if the count is smaller than 50 worms) under a stereoscope.

### Colony formation assay on agarose pads

Agarose pads were generated by molding melted 2% agarose (A9539-500G, Millipore-Sigma) in S Medium between two clean glass plates spaced with 1 mm-thick spacers. We used a 7 mm diameter biopsy punch (Queens Surgical) to cut out discs of agarose pads. The pads were then transferred to a 60 mm petri dish (351007, Corning) with a pair of tweezers with carbon fiber tips (159C-RT, Excelta). Worms of desired number were transferred to the top of the agarose pads. Extra liquid was removed using the tip of a piece of Kimwipe (06–666 A, Kimberly-Clark) twisted between fingers. The dish was then covered and imaged under a DMi8 fluorescence microscope (Leica).

### Colony formation assay on dishes

To make an agarose plate, we dispensed 5 mL 2% agarose with S Medium in a 60 mm petri dish and allowed to solidify. Afterwards, we deposited worms of desired number on to the agarose plate with a pipette. We removed excess liquid and gently dispersed the worms with a soft PVA sponge (40400-8, BVI). The dish was then covered with a lid layered with 2% agarose to reduce condensation and placed on a flatbed scanner (B11B198011, Perfection V600, Epson) for image capture. Scanned photos were taken at 1200 dpi and regular time intervals with a custom Python script.

### Image processing and motion tracking

Before segmentation and tracking, a 2D Gaussian kernel was applied to individual images to reduce local fluctuations. For dauer worms, the outlines of worms and colonies were generated by thresholding on the edge intensities created by convolution with a Roberts kernel. Object masks for worms and colonies were generated by inverting the binary map of a background flood on the thresholded edge map. Object mask openings of size less than 1000 µm^2^ were removed. We tracked objects by finding the nearest centroid Euclidean neighbor in the previous frame. If the nearest neighbor in the last or the next frame was far enough away that a speed greater than 250 µm/sec would have been required to account for the displacement, the object was considered to be an orphan. Depending on the event sequence and the distance to the edge of the field of view, orphans were classified as entering or exiting a colony or entering or exiting the field of view. Given the uniformity of size of dauer worms, objects were categorized into a worm or a colony by its area of mask opening. Objects containing two worms in two consecutive frames, or algorithmically greater than 1.75 times the area of the median opening in the field of view (1.75 times the typical size of a solitary worm) were classified as a colony. Manual inspection was performed to ensure successful implementation.

For adult worms, joining rates and leaving rates were assessed manually, because their sizes were too variable to permit accurate automatic segmentation and tracking.

### Mean square displacement calculations

To calculate the mean square displacement for individual traces, we first took the Cartesian coordinates (*x*, *y*) at the center of mass for an individual worm at each time-step in a trace generated by the tracking algorithm. For each trace, the squared displacements were calculated by summing the square of the differences in *x* and *y* between all pairs of the center of mass coordinates that are separated by a time difference, *t* = *nδt*, where *δt* is the time interval between two consecutive frames and *n* is a positive integer signifying the number of frames separating the two frames. The mean squared displacement for individual traces was calculated by averaging the squared displacements grouped by *t*. The individual mean squared displacements were plotted against *t*. To express this in mathematical terms, .., where *N* is the number of frames in a trajectory and *x* and *y* are the coordinates at *iδt* or *iδt* + *nδt*. Data points from the same trace were connected by straight lines.

We calculated the ensemble mean square displacements similarly, but instead of averaging the squared displacements from individual traces, we calculated the mean of the squared displacements from all traces.

### Cosine-similarity calculation

For individual traces, the velocity of a worm at a particular timepoint was calculated as the translocation divided by the time interval in a 3-second window centered on that timepoint. For every pair of points in a trace, a cosine-similarity value was calculated as (*v*_*i*_·*v*_*j*_)/(|*v*_*i*_|·|*v*_*j*_|), where *v*_*i*_ is the velocity vector at timepoint *i* and *v*_*j*_ is the velocity vector at timepoint *j*. Cosine-similarity values were grouped by the time interval, *τ* = (*i−j*)*δt*, where *δt* is the time interval between two consecutive frames. Cosine-similarity values were also binned by the angle between the worm’s velocity vector and the vector pointing from the center of the nearest in-frame colony to the center of mass of the worm, starting with the 1.5 s frame.

### Reporting summary

Further information on research design is available in the Nature Research Reporting Summary linked to this article.

## Supplementary information

Supplementary Information

Description of Additional Supplementary Files

Supplementary Movie 1

Supplementary Movie 2

Supplementary Movie 3

Supplementary Movie 4

Reporting Summary

## Data Availability

The data that support this study are available from the corresponding author upon reasonable request. The imaging data generated in this study have been deposited in the Stanford Digital Repository at https://purl.stanford.edu/qd784cd0342. The custom code used in the analysis in this study has also been deposited in the Stanford Digital Repository at https://purl.stanford.edu/qd784cd0342. Source data are provided with this paper.

## Source data

Source Data
