# Supplementary material for: C. elegans colony formation as a condensation phenomenon
Source: Nat Commun. 2021 Aug 16;12:4947. doi: 10.1038/s41467-021-25244-9 (PMC8368178; doi:10.1038/s41467-021-25244-9)
Supplement: Supplementary file 1 — Supplementary Information [file 41467_2021_25244_MOESM1_ESM.pdf]

Supplementary Materials for

C. elegans colony formation as a condensation phenomenon

Yuping Chen[1], James E. Ferrell, Jr.[1,2]*
Correspondence to: james.ferrell@stanford.edu

This PDF file includes:

Supplementary Text
Supplementary Figs. 1-3
Captions for Supplementary Movies 1-4

Other Supplementary Materials for this manuscript include the following:

Supplementary Movies 1-4

Supplementary Text

<u>A rate equation model for co-existence of two or more *C. elegans* colonies</u>
In Fig. 1 we observed co-existence of multiple *C. elegans* colonies on the same plate. Here we provide a rate equation model to describe the phenomenon. For simplicity, we first examine a three compartment system consisting of solitary worm (w), colony 1 (w^1*), and colony 2 (w^2*). Similar to the single-colony scenario described in the main text, rate equations for w^1* and w^2* can be written as:

$$\frac{dw^{1*}}{dt} = k_1(w_{tot} - w^{1*} - w^{2*})(w^{1*})^{\frac{1}{2}} - k_{-1}(w^{1*})^{\frac{1}{2}}, \text{ and} \quad (12)$$

$$\frac{dw^{2*}}{dt} = k_1(w_{tot} - w^{1*} - w^{2*})(w^{2*})^{\frac{1}{2}} - k_{-1}(w^{2*})^{\frac{1}{2}}, \quad (13)$$

where we used the conservation relationship $w_{tot} = w + w^{1*} + w^{2*}$.
At steady state, the time derivatives in both Eq. 12 and Eq. 13 must equal zero. There are two solutions:

$$w_{SS}^{1*} = 0 \text{ and } w_{SS}^{2*} = 0, \text{ or} \quad (14)$$

$$w_{SS}^{1*} + w_{SS}^{2*} = w_{tot} - {}^{k_{-1}}/_{k_1}, \quad (15)$$

For $0 \leq w_{tot} \leq k_{-1}/k_1$ there is a single steady state given by Eq 14, and it is stable. When $w_{tot} > k_{-1}/k_1$, there are two steady states given by Eqs 14 and 15. The $w_{SS}^{1*} = w_{SS}^{2*} = 0$ steady state is unstable and corresponds to the dispersed worms being supersaturated. The steady state given by Eq 15 is stable. Note that the total of the worms in colonies 1 and 2 is given by the right hand side, but there is no constraint upon how many worms are in one colony versus the other. This is a consequence of having both the joining and leaving rates be proportional to the colony diameter. As was seen in the single-colony scenario analyzed in the main text, there is a transcritical bifurcation at a threshold of k_{-1}/k_1.

Therefore, the model's predictions are the same irrespective of whether there are one or two colonies. One can apply a similar treatment to systems of colony number greater than two and obtain similar results.

<u>A rate equation model for Ostwald ripening in *C. elegans* colony formation</u>
To account for the Ostwald ripening phenomenon in *C. elegans* colony formation, we suppose that there are three compartments—a small colony (w*), a large colony (w**), and out-of-colony worms (w) (Fig. 5c)—and we assume that large colonies are more stable than small colonies due because worms at the boundary are more exposed when the curvature is higher. We used a function of the form of the Gibbs-Thomson equation[41] to account for this difference in stability:

$$\frac{k_{-1}}{k_1}[r] = \frac{k_{-1}}{k_1}[\infty]e^{a/r}, \quad (16)$$

where a is a parameter that determines how steeply the vapor pressure, chemical potential, or solubility of a substance falls as r increases, in an ideal system. Dividing Eq 6 through by k_1, and substituting in Eq 16 for k_{-1}/k_1, we get:

$$0 = (w_{tot} - w^*)(w^*)^{\frac{1}{2}} - \frac{k_{-1}}{k_1}[\infty]e^{a/(w^*)^{\frac{1}{2}}}(w^*)^{\frac{1}{2}}. \quad (17)$$

Supplementary Fig. S3a, b shows the steady state values of w^* and w as functions of w_{tot}. As was the case with the model shown in Fig. 5c, there is a critical value of w_{tot} below which no colony forms—there is a single steady state with $w^* = 0$. As w_{tot} passes through this value, the system goes through a saddle-node bifurcation, with a stable $w^* > 0$ state appearing and then splitting into a stable and an unstable steady state; the system is bistable. The stable steady state with $w^* = 0$ persists, but it becomes metastable. When $a = 0$, Eq 17 is equivalent to Eq 6, the saddle-node bifurcation reduces to a transcritical bifurcation, and the critical value of $w_{tot} = k_{-1}/k_1$. When $a > 0$, the critical value of w_{tot} becomes greater than k_{-1}/k_1, and the larger the value of a, the greater this discrepancy becomes. As described below, from the experimental agreement between $(w_{tot})_{crit}$ and $w\,\frac{Leaving\ rate}{Joining\ rate}$ we can infer that a is unlikely to be greater than 0.1.

Note also that in this model, the maximum "solubility" of w—the concentration of w when w_{tot} is above the critical value—is no longer constant. Instead w falls as w_{tot} increases, asymptotically approaching a minimal constant value of k_{-1}/k_1. Given experimental variability, this decrease in w with increasing w_{tot} would probably be discernible if a were greater than ~0.2 or 0.3. Since no decrease was observed experimentally (Fig 1e,g), this again argues for a relatively low value of the scaling factor a.

We can derive a formula for the position of the saddle-node bifurcation by noting that it occurs when the steady-state response curve is vertical. First, we write the equation for the relationship between w_{tot} and w^* at steady state. Rearranging Eq 17 yields:

$$w_{tot} = \frac{k_{-1}}{k_1}e^{a/\sqrt{w^*_{SS}}} + w^*_{SS}, \quad (18)$$

for $w^*_{SS} \neq 0$. Note that this equation reduces to Eq 8 when $a = 0$. Next, we take the derivative of w_{tot} with respect to w^*_{SS}:

$$\frac{dw_{tot}}{dw^*_{ss}} = 1 - \frac{ak_{-1}}{2k_1 w^*_{ss}}e^{a/\sqrt{w^*_{SS}}}. \quad (19)$$

When this derivative equals zero, the w^*_{SS} curve is vertical. Setting the derivative to zero produces a transcendental equation, and solving for w^*_{SS} yields:

$$w^*_{SS} = \frac{a}{9ProductLog\left[\frac{1}{3}\left(\frac{2a^2k_1}{k_{-1}}\right)^{2/3}\right]}, \quad (20)$$

where *ProductLog* denotes a Lambert *W* function. The corresponding value for w_{tot} can then be calculated from Eq 18. The critical value of w_{tot} as a function of a, for $k_1 = k_{-1} = 1$, is shown in Supplementary Fig. 3c; over this range, it is nearly a straight line.

Note also that unless a is close to 0, we can no longer obtain the either k_{-1}/k_1 or the critical value of w_{tot} directly from Eq 11. Now we have:

$$w\frac{Leaving\ rate}{Joining\ rate} = \frac{k_{-1}}{k_1}e^{a/\sqrt{\frac{a}{9ProductLog\left[\frac{1}{3}\left(\frac{2a^2k_1}{k_{-1}}\right)^{2/3}\right]}}}. \tag{21}$$

When $a = 0.1$, $w\frac{Leaving\ rate}{Joining\ rate}$ is ~11% below the actual critical value of w_{tot}, and when $a = 1$, it is ~32% low. Since experimentally the critical value of w_{tot} predicted from $w\frac{Leaving\ rate}{Joining\ rate}$ was higher than the observed value for dauer worms, and only ~12% lower for adult worms, the value of a for the worm systems appears to be no larger than 0.1 or so. Thus the effects of curvature appear to be weak.

Now to extend the analysis to three compartments, with a large colony (w^{**}) as well as a small one (w^{*}), we write two rate equations:

$$\frac{dw^*}{dt} = k_1(w_{tot} - w^* - w^{**})(w^*)^{\frac{1}{2}} - k_{-1}[\infty]e^{a/(w^*)^{\frac{1}{2}}}(w^*)^{\frac{1}{2}} \tag{22}$$

$$\frac{dw^{**}}{dt} = k_1(w_{tot} - w^* - w^{**})(w^{**})^{\frac{1}{2}} - k_{-1}[\infty]e^{a/(w^{**})^{\frac{1}{2}}}(w^{**})^{\frac{1}{2}}. \tag{23}$$

The conservation equation now is $w_{tot} = w + w^* + w^{**}$. Note that arbitrarily we have included the Gibbs-Thomson factor, which we used as a correction to k_{-1}/k_1 in the steady-state equation (Eq 17), as being part of the k_{-1} term in these dynamical equations.

In Ostwald ripening, a dominant colony grows and a smaller colony shrinks and eventually vanishes (Supplementary Fig. 3d, e). Although different initial sizes of the two colonies may result in identical steady states — the larger colony dominates and the smaller colony dissolves — the initial dynamics of the process of achieving the steady state may vary greatly. To illustrate the variation in the processes, here we show two examples with different initial conditions. In the first example, both w^* and w^{**} are small, and w^{**} is only slightly greater than w^* (Supplementary Fig. 3d). Both colonies grow initially (also reflected in our experimental observation in Fig. 1) but then diverge, with w^{**} sustains to grow and w^* starts to shrink. The system takes a long time to arrive at the steady state. However, in the second example, where the size of the colonies differs by a greater amount, w** grows and w* dissolves, and the system more quickly transits into the steady-state (Supplementary Fig. 3e).

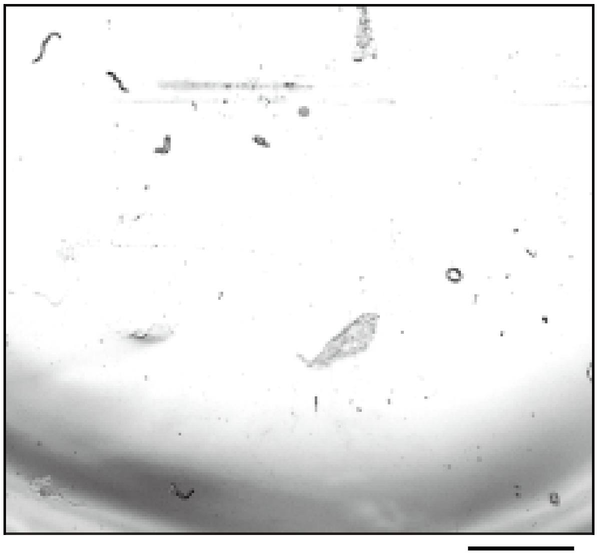

Supplementary Fig. 1: Sparsely seeded worms showed no colonies after 12 h of incubation. Washed adult N2 *C. elegans* seeded at a low concentration (0.01 worms/mm^2) and incubated for 12 h. Scale bar

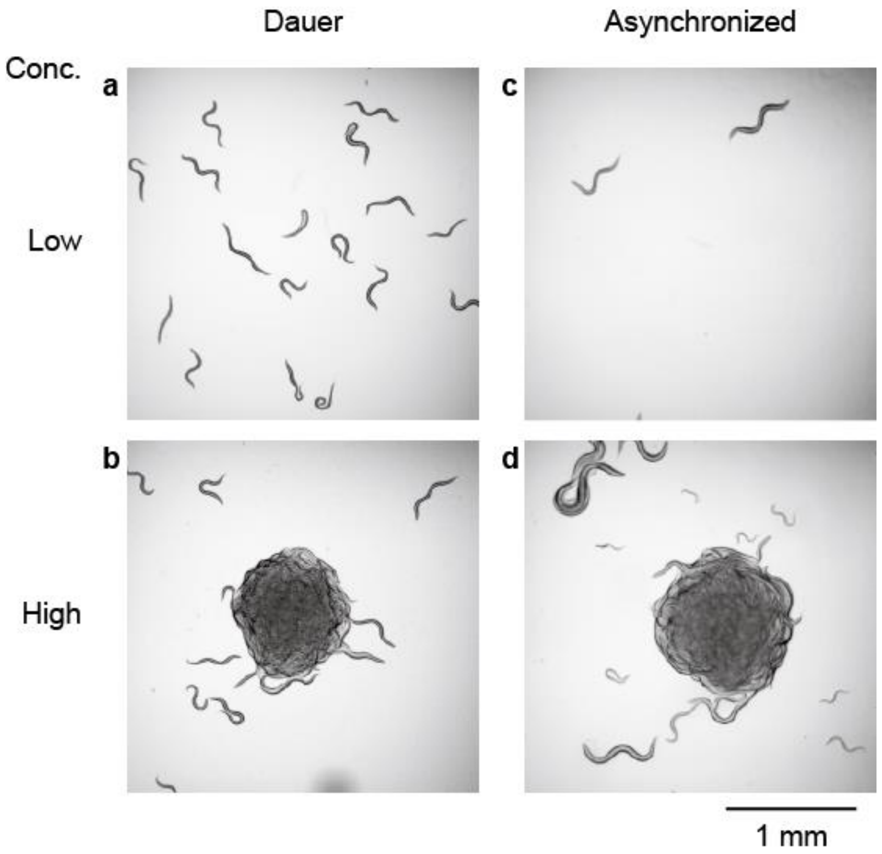

Supplementary Fig. 2: Colony formation in other developmental stages also depends on density.

a, **b** Example micrographs of dauer-stage worms on agarose pads, which (**a**) did not form colonies when the population density was low (0.26 worms/mm^2, n = 7) and (**b**) formed a colony when the population density was high (3.90 worms/mm^2, n = 10). **c**, **d** Example micrographs of asynchronized worms on agarose pads. They (**c**) did not form colonies when the population density was low (0.03 worms/mm^2, n = 9) and (**d**) formed a colony when the population density was high (1.30 worms/mm^2, n = 9).

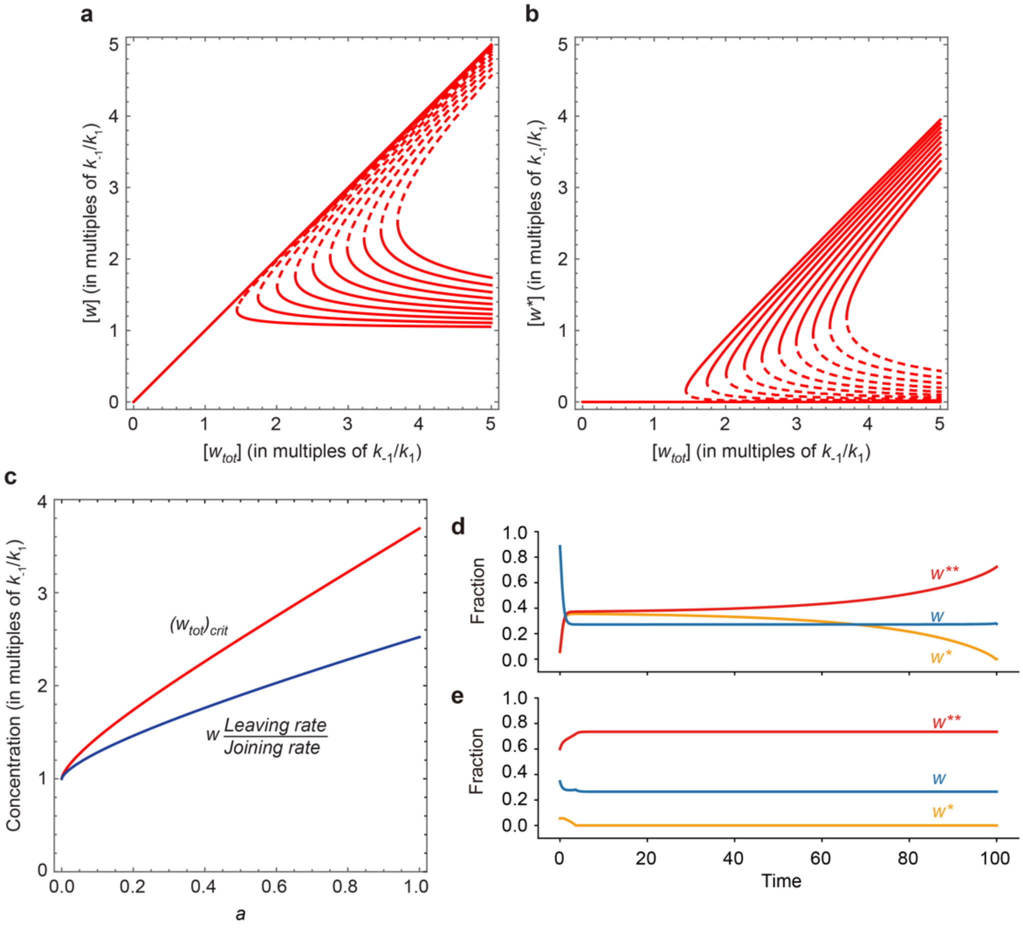

Supplementary Fig. 3: Model for Ostwald ripening in *C. elegans* colony formation.

a, **b** The modeled steady-state densities of worms out of colonies (w), and in a colony (w^*), as a function of the total density of worms w_{tot}, based on numerical solutions to Eq. 15. The system has a single stable steady state until the concentration of worms reaches a critical value that is greater than k_{-1}/k_1. Beyond the critical density, the system undergoes a saddle-node bifurcation, and has an unstable steady state (dashed) and two stable steady states (solid). One stable steady state can be regarded as a supersaturated system; it lies close to the unstable branch, and so small perturbations would be expected to drive the system across the unstable branch and into the stable manifold of the other steady state. **c** Calculated values for the critical concentration of w_{tot} (red) and the concentrations obtained from observation of the joining and leaving rates (blue), as a function of a. **d**, **e** Two examples of modeled time dependent processes based on Eqs. 20 and 21. The parameters for system are: $w_{tot} = 4$, greater than the critical density; k_1 and k_{-1} are both 1; $a = 0.1$, showing size dependency. The initial size of the small colony is $w^*[0] = 0.218$, a small value. We initiated the large colony colony size $w^{**}[0]$ to be either a very large colony ($w^{**}[0] = 2.4$) (**d**) or one similar in size to $w^*[0]$ ($w^{**}[0] = 0.24$) (**e**).
