# Supplementary material for: C. elegans colony formation as a condensation phenomenon
Source: Nat Commun. 2021 Aug 16;12:4947. doi: 10.1038/s41467-021-25244-9 (PMC8368178; doi:10.1038/s41467-021-25244-9)
Supplement: Supplementary file 2 — Description of Additional Supplementary Files [file 41467_2021_25244_MOESM2_ESM.pdf]

Description of Additional Supplementary Files

File Name: Supplementary Movie 1
Description: **Adult colony formation at high density.**
The initial density was 0.18 worms/mm2 . The time stamp is in min:s and the total duration of the movie is 24 min.

File Name: Supplementary Movie 2
Description: **Adult worms at low density showing no colonies.**
The initial density was 0.01 worms/mm2 . The time stamp is in min:s and the total duration of the movie is 24 min.

File Name: Supplementary Movie 3
Description: **Dauer worms dynamically entering and leaving a colony.**
Worms in the colony are false-colored yellow, and individual worms are false-colored blue. The worms' trajectories are initially colored blue and then progressively shift to yellow as time proceeds. Red asterisks denote events where worms entered the colony.

File Name: Supplementary Movie 4
Description: **Mixing of fluorescent and non-fluorescent worms to track single worm dynamics.**
The orange line denotes the boundary of the colony, which is made up mostly of nonfluorescent worms.
