# Supplementary material for: C. elegans colony formation as a condensation phenomenon
Source: Nat Commun. 2021 Aug 16;12:4947. doi: 10.1038/s41467-021-25244-9 (PMC8368178; doi:10.1038/s41467-021-25244-9)
Supplement: Supplementary file 7 — Reporting Summary [file 41467_2021_25244_MOESM7_ESM.pdf]

nature research

Corresponding author(s): James Ferrell

Last updated by author(s): Jul 19, 2021

Reporting Summary

Nature Research wishes to improve the reproducibility of the work that we publish. This form provides structure for consistency and transparency in reporting. For further information on Nature Research policies, see our Editorial Policies and the Editorial Policy Checklist.

Statistics

For all statistical analyses, confirm that the following items are present in the figure legend, table legend, main text, or Methods section.

n/a	Confirmed	
☐	☒	The exact sample size (*n*) for each experimental group/condition, given as a discrete number and unit of measurement
☐	☒	A statement on whether measurements were taken from distinct samples or whether the same sample was measured repeatedly
☐	☒	The statistical test(s) used AND whether they are one- or two-sided<br>*Only common tests should be described solely by name; describe more complex techniques in the Methods section.*
☒	☐	A description of all covariates tested
☒	☐	A description of any assumptions or corrections, such as tests of normality and adjustment for multiple comparisons
☐	☒	A full description of the statistical parameters including central tendency (e.g. means) or other basic estimates (e.g. regression coefficient) AND variation (e.g. standard deviation) or associated estimates of uncertainty (e.g. confidence intervals)
☐	☒	For null hypothesis testing, the test statistic (e.g. *F*, *t*, *r*) with confidence intervals, effect sizes, degrees of freedom and *P* value noted<br>*Give P values as exact values whenever suitable.*
☒	☐	For Bayesian analysis, information on the choice of priors and Markov chain Monte Carlo settings
☒	☐	For hierarchical and complex designs, identification of the appropriate level for tests and full reporting of outcomes
☒	☐	Estimates of effect sizes (e.g. Cohen's *d*, Pearson's *r*), indicating how they were calculated

Our web collection on statistics for biologists contains articles on many of the points above.

Software and code

Policy information about availability of computer code

Data collection	Microscopy data: Leica Application Suite X<br>Flat-bed scanner data: EPSON Scan 3.9.0.0
Data analysis	Image analyses were done with custom code written in Python 3.7.

For manuscripts utilizing custom algorithms or software that are central to the research but not yet described in published literature, software must be made available to editors and reviewers. We strongly encourage code deposition in a community repository (e.g. GitHub). See the Nature Research guidelines for submitting code & software for further information.

Data

Policy information about availability of data

All manuscripts must include a data availability statement. This statement should provide the following information, where applicable:
- Accession codes, unique identifiers, or web links for publicly available datasets
- A list of figures that have associated raw data
- A description of any restrictions on data availability

The data that support the findings of this study are available from the corresponding author upon reasonable request.

Field-specific reporting

Life sciences study design

All studies must disclose on these points even when the disclosure is negative.

Sample size	No sample-size calculation was performed. Data were typically collected from at least three independent experiments. Multiple data points were typically generated from each independent experiment. Hundreds of time series were collected for Fig. 3d.
Data exclusions	No data were excluded from the analyses.
Replication	Data were typically collected from at least three independent experiments. All attempts were successful.
Randomization	Organisms were either not allocated into experimental groups (not relevant), or drawn from the same well-mixed pool with a pipette (randomized).
Blinding	The investigators were not blinded to group allocation during data collection, as it was not possible to perform the experiment without noticing the differences in the population densities of the organisms. The investigators were blinded to group allocation during analysis. Classification decisions were made by computer algorithms.

Reporting for specific materials, systems and methods

We require information from authors about some types of materials, experimental systems and methods used in many studies. Here, indicate whether each material, system or method listed is relevant to your study. If you are not sure if a list item applies to your research, read the appropriate section before selecting a response.

Materials & experimental systems

n/a	Involved in the study	
☒	☐	Antibodies
☒	☐	Eukaryotic cell lines
☒	☐	Palaeontology and archaeology
☐	☒	Animals and other organisms
☒	☐	Human research participants
☒	☐	Clinical data
☒	☐	Dual use research of concern

Methods

n/a	Involved in the study	
☒	☐	ChIP-seq
☒	☐	Flow cytometry
☒	☐	MRI-based neuroimaging

Animals and other organisms

Policy information about studies involving animals; ARRIVE guidelines recommended for reporting animal research

Laboratory animals	Caenorhabditis elegans strains: N2, GA631. Sex: hermaphrodites. Age: 3-20 days.
Wild animals	The study did not involve wild animals.
Field-collected samples	The study did not involve filed-collected samples.
Ethics oversight	No ethical approval or guidance was required. Only invertebrate organisms were involved in the study.

Note that full information on the approval of the study protocol must also be provided in the manuscript.
